# A 4-Year Study of the Association between Short Sleep Duration and Change in Body Mass Index in Japanese Male Workers

**DOI:** 10.2188/jea.JE20100019

**Published:** 2010-09-05

**Authors:** Chihiro Nishiura, Hideki Hashimoto

**Affiliations:** 1 Department of Safety and Health, Tokyo Gas Co, Ltd, Tokyo, Japan; 2 Department of Health Economics and Epidemiology Research, School of Public Health, The University of Tokyo, Tokyo, Japan

**Keywords:** Japanese, longitudinal studies, obesity, sleep, weight gain

## Abstract

**Background:**

Studies of Western populations have shown an inconsistent longitudinal association between short sleep duration and change in body mass index (BMI); a recent Japanese cohort study reported a significant association in men, but over a 1-year period. The aim of this longitudinal study was to examine whether this association was robust over a 4-year interval in Japanese men.

**Methods:**

A total of 3803 middle-aged Japanese male white-collar workers (mean age 47.8 years, mean BMI 23.9 kg/m^2^) in Tokyo, Japan, were included in this study from 1994–1995 (baseline) to 1998–1999 (follow-up). Height and weight were objectively measured at annual health checkups, and other data, including sleep duration, were collected using a structured interview. We used linear regression models to estimate change in BMI, after adjustment for covariates. The reference category for sleep duration was set to 7 hours, to conform with previous studies.

**Results:**

As compared with participants sleeping 7 hours, those sleeping 5 hours or less had a significantly higher BMI at baseline (beta coefficient: 0.34 kg/m^2^, 95% confidence interval (CI): 0.03, 0.65) and gained 0.15 kg/m^2^ in BMI over 4 years (95% CI: 0.03, 0.27), after adjustment for age, baseline BMI, lifestyle behavior, and medication.

**Conclusions:**

The longitudinal association between short sleep duration at baseline and relative increase in BMI was significant in Japanese male workers over a 4-year interval.

## INTRODUCTION

Short sleep duration (5 hours of sleep or less) has been suggested as a risk factor for obesity in adults, although the association between sleep duration and obesity has been criticized as spurious owing to weaknesses in study designs, small sample sizes, and the use of self-reported measurements of body weight and height.^1^^–^^9^ Although recent longitudinal studies have successfully overcome the design weaknesses of earlier studies, the findings remain controversial. Stranges et al observed a large white occupational cohort for 5 years and found no significant relationship between self-reported short sleep duration and increase in body mass index (BMI) among men or women.^10^ In a recent study, Watanabe et al observed a large Japanese occupational cohort for 1 year, and reported a significant increase in BMI among male, but not female, self-reported short sleepers.^11^

It is difficult to easily compare these studies, because of differences in both race and the length of the follow-up period. The question as to whether a longitudinal association between short sleep duration and increase in BMI would be observed in a Japanese cohort after a follow-up period longer than 1 year deserves further epidemiological inquiry. A long-term follow-up study by Patel et al found that the relative increase in mean weight change in short sleepers was greatest after a 4-year interval, during a total follow-up period of 16 years.^6^ Thanks to the inclusion of a very large cohort, Watanabe et al successfully identified a significant, yet very small, beta coefficient (0.013 kg/m^2^ per year)^11^ over a 1-year observation period. If a larger coefficient were observed after a 4-year interval, it would support the hypothetical link between short sleep duration and increased BMI among Japanese male workers.

In this study, we aimed to examine the lasting effect of short sleep duration at baseline on increase in BMI after a 4-year follow-up in a large sample of Japanese male workers.

## METHODS

### Study population

The present data were derived from annual health checkups conducted at a gas company in Tokyo, Japan from October 1994 to September 1995 (baseline survey) and from October 1998 to September 1999 (follow-up survey) in a 4-year period. The company employees were almost all white-collar workers. Because Japanese law mandates that every employer offer an annual health checkup to its employees, the actual participation rate was 98.1% at the baseline survey and 97.8% at the follow-up survey. At the baseline checkup, the company offered employees aged 40–59 years a lifestyle questionnaire, which included items on sleep duration. This questionnaire was the data source of our current study. Available data were limited to men because the company did not provide information on women, due to the small number of female employees and concerns regarding confidentiality. The total number of male workers in their 40s and 50s was 4826 at the baseline survey. After exclusion for failed checkups (*n* = 142) and missing responses for key variables (*n* = 240), 4444 respondents who provided complete data during the baseline survey were included. Of these, 3803 respondents (response rate, 79% of the 4826 subjects) were available after excluding those lost to follow-up (*n* = 641). As compared with the 3803 remaining subjects that were included in the analysis, the 240 subjects with missing questionnaire responses were significantly older (mean 49.3 years vs. 47.8 years), but did not differ in BMI. As compared with those included in the analysis, those lost to follow-up were significantly older (mean 53.6 years vs. 47.8 years), less likely to smoke (51.0% vs. 56.3%), less likely to have a family history of diabetes (11.9% vs. 15.0%), and more likely to take current medication for hypertension (13.1% vs. 8.5%). They did not differ in any other variables, including BMI and sleep duration.

### Dependent variables

At the baseline and follow-up surveys, the height and weight of each participant were objectively measured by trained health professionals. BMI was calculated as weight in kilograms divided by the square of height in meters.

### Independent variables

All covariates except age were derived from a structured interview conducted by trained health professionals at the baseline survey; age was determined using the company’s administrative records. The data for sleep duration were collected by asking “How many hours, on average, do you sleep each night?” and then further categorized into 4 groups: 5 hours or less, 6 hours, 7 hours, and 8 hours or more of sleep. The reference category of sleep duration was set to 7 hours, to permit comparison with previous studies.^5^^,^^6^^,^^9^^–^^11^ The other covariates were baseline height (for the cross-sectional model only, continuous) or baseline BMI (for the longitudinal model only, continuous), current medications (none; 1 or more medications for diabetes, dyslipidemia, hypertension, or psychiatric illness), family history of disease (none; 1 or more of family history of diabetes, dyslipidemia, or hypertension), smoking status (current smoker, nonsmoker), habitual alcohol consumption (defined as consuming alcohol at least once per week), and habitual exercise (defined as exercising at least once per week).

### Statistical analysis

Baseline differences between sleep duration categories were assessed using analysis of variance with the Scheffé post-hoc test for continuous variables, and the chi-square test with the cellwise post-hoc test for categorical variables. The statistical significance of cells in the contingency tables was estimated by using the adjusted residuals. The cells were considered significant when the adjusted residuals were greater than 2. Cross-sectional and longitudinal relationships between sleep duration and BMI were examined by multivariate linear regression models for BMI at baseline and change from baseline BMI at follow-up. Regression models consisted of an age-adjusted model (adjustment only for age) and a fully adjusted model (including all of the abovementioned covariates). Further analysis of the longitudinal relationship stratified by baseline BMI tertiles (<22.70, 22.70–24.84, and ≥24.85 kg/m^2^) was conducted to estimate changes in BMI (kg/m^2^) plus 95% confidence intervals after adjusting for covariates. Effect modification was assessed by testing interaction terms in the regression models, which were remodeled by treating the continuous values of sleep duration instead of the categorized ones. All statistical analyses were carried out using SPSS version 15.0J software (SPSS Inc., Chicago, IL, USA).

### Ethical approval

The company’s authority approved the secondary use of the health checkup data for this study, and oral consent for the generic use of the health checkup data for research purposes was obtained from all participants at the time of the checkups. All data were provided anonymously.

## RESULTS

### Cross-sectional association between sleep duration and BMI at baseline

The baseline characteristics of the participants are shown in Table 1. Regarding the proportions of participants in the sleep duration categories, 8.9% slept 5 hours or less, 42.6% slept 6 hours, 39.2% slept 7 hours, and 9.3% slept for 8 hours or more. Only 21 respondents (0.6%) slept 9 hours or more (data not shown); they were included in the last category. Despite similar height distributions, shorter sleepers were significantly more likely to have a higher BMI at baseline. The Scheffé post-hoc test showed a significance difference in BMI at baseline between those sleeping 6 hours and those sleeping 7 hours (*P* = 0.03). Long sleepers (≥8 hours) tended to be older (*P* < 0.01 for ≥8 hours vs. 6 hours, and *P* = 0.04 for ≥8 hours vs. 7 hours, by Scheffé post-hoc test) and to take medication (significant difference in prevalence of hypertension, psychiatric illness, and 1 or more current medications, by cellwise post-hoc test). After adjustment for covariates, baseline BMI was significantly higher among the short sleeper groups (Table 2, upper rows).

**Table 1.** Baseline characteristics^a^ and changes in weight/BMI of participants by sleep duration (*n* = 3803)

	Overall	Self-reported sleep duration (hours)				*P* value^b^

		≤5	6	7	≥8	
No. of subjects	3803	338	1622	1491	352	
Age (years)	47.8 (5.3)	48.1 (5.5)	47.5 (5.1)	47.8 (5.3)	48.7 (5.6)	<0.001
Height (cm)	168.6 (5.5)	168.5 (5.8)	168.6 (5.5)	168.6 (5.5)	168.3 (5.6)	0.800
Weight (kg)	67.8 (8.7)	68.3 (8.2)	68.3 (8.8)	67.4 (8.7)	66.9 (9.0)	0.007
BMI (kg/m^2^)	23.9 (2.7)	24.0 (2.4)	24.0 (2.7)	23.7 (2.7)	23.6 (2.8)	0.003
Current medication						
Diabetes (%)	2.1	2.4	2.6	1.6	1.4	0.205
Dyslipidemia (%)	1.6	1.8	1.4	1.5	2.3	0.692
Hypertension (%)	8.5	8.0	8.1	8.0	12.8	0.027
Psychiatric illness (%)	0.2	0.0	0.2	0.1	1.1	0.003
≥1 of the above (%)	11.6	11.2	11.2	10.7	17.0	0.009
Family history of disease						
Diabetes (%)	15.0	14.8	15.8	14.0	16.2	0.481
Dyslipidemia (%)	0.6	0.6	0.4	0.7	0.6	0.737
Hypertension (%)	35.3	34.0	36.6	33.3	38.6	0.126
≥1 of the above (%)	43.7	40.8	45.1	42.3	46.3	0.203
Drinking habit (%)	83.2	81.1	83.4	83.2	84.1	0.723
Exercise habit (%)	36.4	32.2	37.7	36.6	34.1	0.215
Smoking habit (%)	56.3	55.6	55.5	56.3	60.5	0.386
ΔWeight (kg)^c^	−0.6 (3.0)	−0.2 (3.0)	−0.7 (3.1)	−0.6 (2.9)	−1.0 (3.0)	0.014
ΔBMI (kg/m^2^)^d^	−0.2 (1.0)	−0.1 (1.0)	−0.2 (1.1)	−0.2 (1.0)	−0.3 (1.0)	0.013

Abbreviation: BMI, body mass index.

^a^Data are presented as means (standard deviation) or percentages.

^b^Statistical significance was assessed by analysis of variance for continuous variables and by the chi-square test for categorical variables.

^c^ΔWeight was calculated by subtracting weight at baseline from weight at follow-up.

^d^ΔBMI was calculated by subtracting BMI at baseline from BMI at follow-up.

**Table 2.** Cross-sectional and longitudinal relationships between sleep duration and BMI (*n* = 3803)

	Self-reported sleep duration (hours)						
	
	≤5		6		7	≥8	
				
	B	95% CI	B	95% CI	Reference	B	95% CI
BMI (kg/m^2^) at baseline (cross-sectional)							
Age-adjusted model	0.34	(0.02, 0.65)	0.29	(0.10, 0.45)	0.00	−0.09	(−0.40, 0.22)
Fully adjusted model^a^	0.34	(0.03, 0.65)	0.27	(0.08, 0.45)	0.00	−0.14	(−0.45, 0.16)
Change in BMI (kg/m^2^) (longitudinal)							
Age-adjusted model	0.13	(0.01, 0.25)	−0.03	(−0.10, 0.04)	0.00	−0.11	(−0.23, 0.01)
Fully adjusted model^b^	0.15	(0.03, 0.27)	−0.01	(−0.08, 0.06)	0.00	−0.11	(−0.23, 0.01)

Abbreviations: BMI, body mass index; CI, confidence interval.

^a^Adjusted for age, current medications, drinking, exercise, family history of disease, height, and smoking.

^b^Adjusted for age, baseline BMI, current medications, drinking, exercise, family history of disease, and smoking.

### Longitudinal association between sleep duration and change in BMI

The overall population showed a decrease in BMI over the study period; shorter sleepers had less of a decrease than the longer sleepers (Table 1, last 2 rows), and the Scheffé post-hoc test revealed a significant difference between those sleeping 5 hours or less and those sleeping 8 hours or more (*P* = 0.02).

Over the follow-up period of 4 years, respondents sleeping 5 hours or less showed significant relative increases in BMI (beta coefficient = 0.13 kg/m^2^, 95% CI: 0.01, 0.25), as compared with those sleeping 7 hours, after adjustment for age (Table 2, lower rows). The beta coefficient slightly increased after adjustment for all covariates.

The Figure shows the longitudinal association between sleep duration and mean change in BMI for baseline BMI tertiles (BMI <22.70, 22.70–24.84, and ≥24.85 kg/m^2^), after adjustment for covariates. These 3 subgroups showed a similar downward gradient in the longitudinal association between sleep duration and change in BMI. Those in the lowest BMI subgroup (<22.70 kg/m^2^) tended to have a larger absolute gain than those in the higher BMI subgroups. There was no effect modification by baseline BMI on the association between sleep duration and change in BMI (log likelihood ratio test; G = 1.019, d.f. = 1, *P* = 0.313) in this sample.

**Figure.**
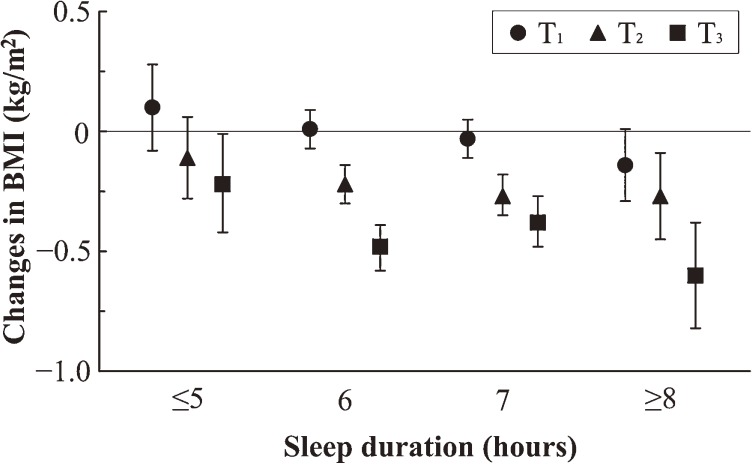
The longitudinal relationship between baseline sleep duration and mean change in BMI over a 4-year interval, by baseline BMI tertiles. The symbols show the mean changes in BMI and the error bars show the 95% confidence interval, after adjustment for age, baseline BMI, current medications, drinking, exercise, family history of disease, and smoking. The tertile values of baseline BMI were: <22.70 kg/m^2^ (T_1_; *n* = 1268), 22.70–24.84 kg/m^2^ (T_2_; *n* = 1270), and ≥24.85 kg/m^2^ (T_3_; *n* = 1265). Abbreviation: BMI, body mass index.

## DISCUSSION

The present analysis of a large occupational cohort of Japanese male workers demonstrated that self-reported short sleep duration was significantly associated with relative increase in BMI over a 4-year interval.

The cross-sectional findings in the present study identified a significant sleep–BMI relationship, as was observed in most previous studies. Stranges et al reported a significant cross-sectional relationship between sleep duration and BMI, but noted no longitudinal relationship. They argued that the cross-sectional association between short sleep duration and change in BMI was not causal, but rather was confounded by comorbid conditions related to obesity, such as sleep apnea and use of anti-depressant medication.^10^ By contrast, the present study found a significant association in both the cross-sectional and longitudinal designs.

Recently, Watanabe et al detected a significant but very small longitudinal sleep–BMI association during a 1-year observation of Japanese male workers.^11^ Our findings extend their results to a 4-year observation period. The beta coefficient for the relative increase in BMI over a 4-year interval among short sleepers (0.15 kg/m^2^ per 4 years) was larger than the 1-year value reported by Watanabe et al (0.01 kg/m^2^ per 1 year). These findings are consistent with those of Patel et al, which showed that the largest mean weight gain was observed after a 4-year follow-up (0.41 kg per 4 years).^6^

Stranges et al did not detect a longitudinal association between short sleep duration and change in BMI over a 5-year follow-up in a white population.^10^ Their subjects were male white-collar workers and were participants in the Whitehall II study; their average age was older, and the proportion of obese subjects was larger, than in our cohort and the cohort of Watanabe et al. These differences in the distributions of age and obesity could be due to the impact of a different, unmeasured, confounder on change in BMI, such as a physiological, behavioral, or genetic condition, which may explain the difference between the findings of Stranges et al and those of the Japanese studies.

As for a non-white population, Lauderdale et al conducted a study that over-sampled an African-American population and, again, did not detect an association.^9^ A simple comparison of the data from Lauderdale et al with the Japanese data, including ours, is not plausible because of the small sample size of the former and the different distribution in socioeconomic status. For instance, Lauderdale et al included unemployed and part-time workers, which could have led to residual confounding due to stress and dietary behavior. Comparisons among studies of participants of the same race and sociocultural background would lead to more credible conclusions on the impact of sleep on BMI. Thus, we believe that the findings of Stranges et al and Lauderdale et al cannot be regarded as counter evidence to our results, and those of Watanabe et al, regarding Japanese male workers.

Although the mechanism for the relationship between short sleep duration and the relative increase in BMI is unclear, some plausible pathways have been suggested, such as stress (the corticoadrenal pathway),^12^ behavioral changes (eg, dietary preference and intake),^13^ and sleep-related neurochemicals (eg, leptin)^14^ and consequent appetite changes.^15^ Studies of Japanese workers also reported that short sleep duration was related to depressive mood^16^ and irregular meal patterns.^17^ Since the present study failed to measure these intermediate factors, the identification of a mediating mechanism is left to future studies.

The strengths of this study include its prospective design, objectively measured BMI, relatively large sample, and the extended measurement of possible confounders such as obesogenic medications. However, this study has several limitations that warrant discussion. First, the study population was restricted to middle-aged Japanese male workers from a single company, which may limit the generalizability of the findings because of socioeconomic differences such as marital status, families living together, occupation, and the company's policies regarding employee health. In fact, age- and sex-based differences in sleep disturbance were reported among Japanese workers.^18^ Additionally, it should be mentioned that the average body weight decreased in the study population over the 4-year period, unlike observations in previous studies.^9^^,^^11^ For instance, Watanabe et al reported that Japanese male workers in the shortest sleep category had an absolute increase in BMI of 0.07 kg/m^2^ over a 1-year observation period.^11^ In the current study, the health staff in the company had for decades provided all employees with personal consultations on diet and exercise after their annual health checkup, and occasionally with mass lectures on health risks. Thus, the decrease in mean BMI in the study sample may have resulted from the company’s general policy regarding health consciousness-raising among employees. In spite of these unique characteristics of the studied subjects, a differential relative change in BMI across sleep duration categories was observed, which was consistent with previous studies and supports an epidemiological association between sleep duration and BMI change. Further research on the wider population may be required to extend the generalizability of our findings. A second limitation of this study was that sleep duration was dependent on self-report, so that differential misclassification of sleep duration might have occurred. Lauderdale et al reported that obese subjects systematically reported a shorter sleep duration than did non-obese subjects with the same objectively measured sleep duration.^19^ Thus, in the cross-sectional observations, our results might be affected by differential misclassification. If differential misclassification arises in longitudinal observations, the longitudinal trend between sleep duration and change in BMI would be expected to vary across the baseline BMI levels. We conducted further analysis stratified by baseline BMI and observed the same trend independent of baseline BMI levels, as shown in the Figure. In addition, we did not detect an effect modification by baseline BMI on the association between sleep duration and change in BMI. For this reason, we believe that the use of self-reported sleep duration did not affect our conclusions. Third, the questionnaire on sleep duration used in this study simply asked for the overall average duration, although Watanabe et al measured sleep duration separately on weekdays and holidays,^11^ which might result in a difference in the beta coefficient values. In spite of the different measures, Watanabe et al and our study both found significant longitudinal sleep–BMI relationships, indicating that short sleep duration is associated with relative increases in BMI. Fourth, we adjusted for the frequency of drinking and exercise without information on the quality of these activities, such as units of alcohol and metabolic equivalents. Although this may cause residual confounding, a previous study with improved adjustments still observed a sleep–weight association.^6^ Finally, since we measured sleep duration only at baseline, we could not distinguish between the lasting effect of short sleep duration on relative increase in BMI and other lasting factors related to baseline sleep duration, such as the level of psychological stress and related behavioral factors. Many previous studies measured sleep duration and stress, but only once and simultaneously at baseline. Since these 2 variables were correlated,^10^^–^^12^ it is difficult to determine which is causal and has a greater influence on change in BMI. Future longitudinal studies with repeated measurements of sleep duration and stress, and with stratified analysis by stress level, might clarify these issues.

In conclusion, short sleep duration at baseline was associated with a relative increase in BMI among Japanese male workers after a 4-year interval. Further research is warranted to determine whether psychological stress, lifestyle behavior, and/or the presence of neurochemicals related to short sleep duration would affect this association and whether this association can be generalized to populations other than middle-aged Japanese male workers.
